# Moxibustion combined with characteristic lifestyle intervention of Traditional Chinese Medicine in the treatment of abdominal obesity: A study protocol for a randomized controlled trial

**DOI:** 10.1097/MD.0000000000022855

**Published:** 2020-10-23

**Authors:** Li-Hua Wang, Wei Huang, Wei Zhou, Li Zhou, Xiao-Li Zhou, Peng Zhou, Yan Yan, Zhong-Yu Zhou, Hua Wang

**Affiliations:** a College of Acupuncture and Orthopedics, Hubei University of Chinese Medicine/Hubei Provincial Collaborative Innovation Center of Preventive Treatment by Acupuncture and Moxibustion; b Department of Acupuncture, Hubei Provincial Hospital of Traditional Chinese Medicine; c Department of Acupuncture, Wuhan Hospital of Traditional Chinese and Western Medicine, Wuhan; d Department of rheumatology, Shanxi Provincial Hospital of Traditional Chinese Medicine, Shanxi; e Department of Acupuncture, Shenzhen Bao’an Traditional Chinese Medicine Hospital Group, Shenzhen; f Department of Acupuncture, Xiangyang Hospital of Traditional Chinese Medicine, Wuhan, China.

**Keywords:** a randomized controlled trial, abdominal obesity, characteristic lifestyle intervention, moxibustion, protocol

## Abstract

**Background:**

Abdominal obesity occurs when excessive visceral and subcutaneous fat is built up around the abdomen and stomach, which negatively impacts human health. Moxibustion, arose from Traditional Chinese Medicine (TCM), has been widely applied in the treatment of abdominal obesity. Several studies have shown the positive effects of moxibustion in prevention and treatment of endocrine issues and excess body weight. In this context, our study aims to examine the safety and efficacy of the combination of moxibustion and characteristic lifestyle intervention of TCM in the treatment of abdominal obesity.

**Methods/design:**

This study will be a multicenter, randomized, controlled trial conducted from September 2020 to January 2022 that includes 150 participants who have abdominal obesity and meet the eligibility criteria. The participants will be randomly divided into 3 groups in a 2:2:1 allocation ratio. The intervention group will receive moxibustion combined with characteristic lifestyle intervention of TCM; the other group will receive moxibustion combined with lifestyle intervention; the control group will receive lifestyle intervention only. Eight-week moxibustion sessions will be provided to participants assigned to the 2 intervention groups. The characteristic lifestyle intervention of TCM will also last 8 weeks, whereas the lifestyle intervention will last 12 weeks including 8-week treatment period, 4-week follow-up period. The primary outcome is the waist circumference measured by a tape measure. The secondary outcomes include obesity-related indicators, serum biochemical indexs, blood pressure, conversion score of physical symptoms, and measurement of the scale. Adverse events will be recorded during the treatment and follow-up period.

**Discussion:**

The results are expected to provide clinical evidence for the application of the combination of moxibustion and characteristic lifestyle intervention of TCM in patients with abdominal obesity.

**Trial registration:**

ClinicalTrials.gov, NCT04501198, Registered on 9 June 2020.

## 1 Introduction

Obesity is a growing global health concern representing an enormous economic and health burden.^[1]^ Excess fat accumulation in the body has shown to be an important risk factor for obesity which can contribute to serious health problems, such as cardiovascular disease, type 2 diabetes mellitus (T2DM), neurodegenerative diseases, liver disease, and even cancer, in particular Alzheimer disease.^[2–4]^ Approximately 650 million people are affected by obesity globally, and this number is estimated to rise in the future.^[5]^ In Iran, the age-adjusted prevalence of overweight or obesity is predicted to be 57.0% in women and 42.8% in men.^[6]^ Moreover, previous studies have confirmed a close association between obesity and psychological problems like anxiety, depression, or stress.^[7]^ It has been shown that nonabdominal obesity was associated with a relatively low risk for mortality and cardiovascular disease, whereas abdominal obesity had higher adverse effects overall, and nonabdominal obesity sometimes even elicited protective effects.^[8]^ In this context, abdominal obesity was a better biomarker to predict mortality than nonabdominal obesity.^[9]^

Nowadays, major clinical treatments of abdominal obesity mainly focus on lifestyle changes (exercise, diet control), drug therapy, surgical treatment, Traditional Chinese Medicine (TCM) intervention, cognition education, among others. The interventions such as diet, physical activity, and cognition education have shown short-term weight loss effects, but poor long-term results and adherence rates.^[10–12]^ In addition, drug therapy is always difficult to be accepted by the majority of obese patients based on its safety. Recent research reported that the surgical treatment of gastrointestinal surgery is the fastest way to lose weight in clinical practice.^[13,14]^ However, only approximately 61.7% population reached the therapeutic goal of weight loss for that the cost and risk of postoperative complications are both high, and there are strict indications, which was unsatisfactory.^[15]^ Therefore, alternative treatment modalities for abdominal obesity are in high demand.

Moxibustion, arose from TCM, has been widely applied in clinical practice. A number of studies have demonstrated that moxibustion is effective in enhancing the immune system, and modifying neurotransmitter levels of central nervous system through repeated acupoint stimulation.^[16,17]^ Studies have shown that moxibustion induces a smoother flow of qi and blood in the human body by using moxa to warm acupoints and body regions, accelerates the blood circulation overall, thereby achieving the effects of disease prevention and health care.^[18]^ Moxibustion can be applied to patients indirectly (placing a medium between the skin and the burning moxa) or directly (placing burn cones directly onto the skin). Moxibustion may be a good choice with patients and doctors for its unique advantages of effectiveness, convenience, and simplicity.

Thus far, moxibustion interventions have been studied in both clinical trials and animal models. Several studies in animal models have shown the positive effects of moxibustion in prevention and treatment of endocrine issues and excess body weight. For example, some researches have proved that moxibustion had beneficial effects on blood lipids, fat accumulation, and decrease of adrenocorticotrophic hormone which plays a key role in decreasing blood flow into fat tissue in rats.^[19,20]^ In one previous clinical trial, Mestre et al found that obesity index decreased and the rate of body weight gain was slowed after the moxibustion treatment.^[21]^ Another study conducted by Huang et al^[22]^ showed that moxibustion was effective in reducing blood lipid indexes and the levels of Leptin, Resistin for the patients with obesity complicated with hyperlipidemia. All of these results suggest that moxibustion has beneficial effects on both body weight management and blood lipids.

To evaluate the exact effect of moxibustion on the clinical efficacy of abdominal obesity, we will conduct a 3three-arm parallel clinical trial. In this study, we will try to combine the advantages of moxibustion and characteristic lifestyle intervention of TCM in the treatment of abdominal obesity. To achieve this, a standardized multicenter, randomized, controlled trial will be carried out to improve the treatment protocol and evaluation criteria, which can provide an objective theoretical basis and methodology for the treatment of abdominal obesity by moxibustion combined with characteristic lifestyle intervention of TCM.

## 2 Methods/design

### 2.1 Ethics approval

This study was approved by the Ethics Committee of Hubei Provincial Hospital of TCM, HBZY2020-C36–01. The recruitment did not start at other centers in this trial until the local ethical approval had been obtained. This study is registered at https://register.clinicaltrials.gov/prs/app/action/LoginUser?ts=1&cx=-jg9qo4(NCT04501198). Written informed consents will be provided by all participants before they were enrolled in this study. Their personal information will be collected, and maintained in an independent closet to protect confidentiality before, during, and after the clinical trial.

### 2.2 Study design

**Figure 1:**
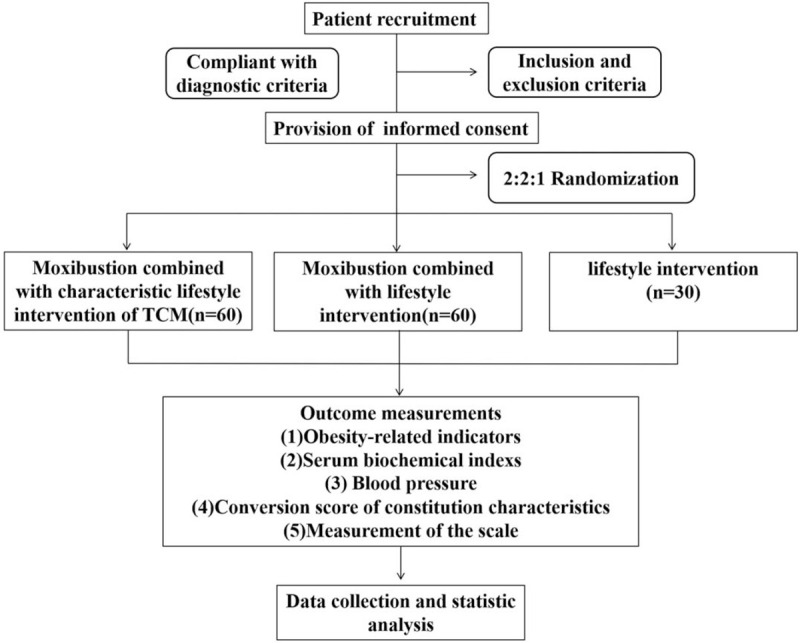
Flowchart of the trial.

This randomized controlled trial will be conducted at the Department of Acupuncture of five hospitals including Hubei Provincial Hospital of Traditional Chinese Medicine, Shanxi Provincial Hospital of Traditional Chinese Medicine, Shenzhen Bao’an Traditional Chinese Medicine Hospital Group, Wuhan Hospital of Traditional Chinese and Western Medicine, Xiangyang Hospital of Traditional Chinese Medicine from September 2020 to January 2022. The participants recruited from our outpatient departments will be randomly allocated to three groups, these are the intervention group (moxibustion combined with characteristic lifestyle intervention of TCM, n = 60,expected); the other intervention group (moxibustion combined with lifestyle intervention, n = 60,expected) or the control group (lifestyle intervention, n = 30, expected). The participants in the 2two intervention groups will receive 48 sessions of moxibustion treatment over 8 weeks. The characteristic lifestyle intervention of TCM will also last 8 weeks, whereas the lifestyle intervention will last 12 weeks including 8-week treatment period, 4-week follow-up period. The study design is depicted in Figure 1.

### 2.3 Participants

#### 2.3.1 Diagnostic criteria

According to the criteria in the Comprehensive Medical Management Guidelines for Obese People jointly issued by the American Society of Clinical Endocrinologists (AACE) and the American College of Endocrinology (ACE) in May 2016:^[23]^ waist circumference (WC) ≥85 cm for male and 80 cm for female can be diagnosed as abdominal obesity.

#### 2.3.2 Inclusion criteria

The inclusion criteria for the participants are as follows:

1. Satisfying the criteria for the diagnosis of abdominal obesity;
2. 18 years’ old≤age≤55 years’ old;
3. Being able to fully understand and voluntarily sign informed consent.

#### 2.3.3 Exclusion criteria

The exclusion criteria for the participants consist of:

1. Presence of endocrine disorders such as: polycystic ovary syndrome; Cushing syndrome; uncorrected thyroid disease.
2. Presence of diabetes mellitus, or hypertension, or abnormal liver and kidney functions, or mental diseases.
3. Pregnant or lactating state, women who plan to become pregnant within 12 weeks.
4. History of bulimia, anorexia, or any other eating disorders.
5. Use of medications in the past 3 months, such as diet drugs, corticosteroids, antidepressants, which may affect weight or appetite.
6. History of surgical weight loss, postoperative adhesions.
7. History of participating in a clinical study of weight loss or any other therapies to lose weight in the past 3 months.
8. Presence of local skin rupture, allergy, and scar constitution.
9. Unable to cooperate with the research caused by other diseases or reasons.

#### 2.3.4 Recruitment

All participants will be recruited by clinicians with a medical practitioner qualification certificate in the outpatient clinics of these 5 participating hospitals. Recruitment methods including hospital website, hospital Public Accounts, and local advertising which will be put up on campus or communities.

#### 2.3.5 Patient safety

Potential adverse events associated with moxibustion included rash, skin allergy, and infection. They will be recorded by the acupuncturist clinicians when any adverse events occur, and the intervention will be immediately stopped but temporarily. Three experts from different fields from Hubei Provincial Hospital of TCM compose an independent Safety Monitoring Board, who has the right to make the final decision to terminate the study. All these experts will monitor the safety and performance of this trial to make sure that the study goes smoothly.

### 2.4 Intervention

After baseline measurements, patients satisfying the inclusion criteria will be randomly divided into 1 of the 3 treatment groups, as shown below:

1. Group A: Lifestyle intervention.
2. Group B: Moxibustion combined with lifestyle intervention.
3. Group C: Moxibustion combined with characteristic lifestyle intervention of TCM.

All the participants will be instructed to return to visit the doctor every 2 week.

#### 2.4.1 Group A (lifestyle intervention)

The following living habits are recommended:^[24]^

All participants who weigh 113.6kg (250 lb.) will be prescribed a diet of 1200 to 1499 kcal/day, which comprise of conventional foods with approximately 20% to 35% from fat, 15% to 20% kcal from protein, and the remainder from carbohydrate. Whereas for those who weighed≥113.6 kg, a diet of 1500 to 1800 kcal/day will be prescribed. Meanwhile, all participants will be instructed to record their food and calorie intake daily, and low-to-moderate intensity physical activities such as jogging and walking will be instructed to them for at least 5 days per week continually, and for at least 210 min/week, preferably ≥270 min/week.

All patients will be instructed to keep a diet and exercise diary.

The intervention time of lifestyle intervention includes the treatment period of 8 weeks and the follow-up period of 4 weeks.

#### 2.4.2 Group B (Moxibustion combined with lifestyle intervention)

##### 2.4.2.1 Moxibustion

The moxibustion acupoints will be selected as ①*zhongwan, guanyuan, shenque,* and *zusanli*; ②*pishu,shenshu.* The location of the acupoints was based on the national GB-12346-90 acupoints standard.^[25]^ Acupoints group ① will be selected on Monday, Wednesday, and Friday, Acupoints group ② will be selected on Tuesday, Thursday, Saturday. The lit moxa (moxa cone of 1.5 cm in diameter, planted in Nanyang, China) was placed 2 to 3 cm above the skin, and the surface temperature of the local skin will be maintained at 45°C ± 2 °C for 20 minutes. Moxibustion treatment will be applied once every day, 6 times per week, for 8 consecutive weeks. The treatment will be delayed during the menstrual period.

##### 2.4.2.2 Lifestyle intervention

The lifestyle intervention in this group will be the same as that for the group A.

#### 2.4.3 Group C (Moxibustion combined with characteristic lifestyle intervention of TCM)

##### 2.4.3.1 Moxibustion

The specific methods and intervention time in this group will be the same as that for the group B.

##### 2.4.3.2 Characteristic lifestyle intervention of TCM

In this study, xiusheng decoction, traditional exercises, and modern lifestyle intervention will be combined as the characteristic lifestyle intervention method of TCM to help participants establish healthy living habits.

(1) Xiusheng Decoction
  The package (specification: 6 g) manufactured by hubei ji’antang pharmaceutical Co., Ltd. is adopted, the manufacturer conforms to GMP standard with strict quality control. The components of this product are all Chinese medicines with medicinal and food homologous (composition: coix seed, black tea, tuckahoe, lotus leaf, xylitol, dextrin), and the dosage form is granule. Each decoction should be taken twice a day, orally half an hour after meals. The treatment will be delayed during the menstrual period. The course of intervention will last for 8 weeks.
(2) Traditional exercises
  It is recommended that the “fitness qigong • ba duan jin” issued by the fitness qigong management center of general administration of sport of China be taken as the standard for performing meritorious exercises. A full set of fitness qigong consists of 8 forms in which the essentials of these movements refer to “Chinese Traditional Health sports and Health Preservation,” and the movements will be instructed by the extension agents of traditional sports who have been trained by Shanghai qigong research institute. The exercise will be taken for 35∼40 minutes every day, 7 times a week. The intervention will be conducted for 8 weeks.
(3) Modern lifestyle intervention
  The lifestyle intervention in this group will be the same as that for the group A.

### 2.5 Outcome measurements

#### 2.5.1 Primary outcome measures

WC will be used as the primary outcome measure. This index will be assessed on week 0, 2,4,6, 8, and 12.

#### 2.5.2 Secondary outcome measures

##### 2.5.2.1 Obesity-related indicators

Obesity-related indicators are weight (WG); body mass index (weight/height); body fat percentage (F%); basic metabolism value; hip circumference (HC); and waist-to-hip ratio (WC/hip circumference) measured on week 0, 2,4,6, 8, and 12.

##### 2.5.2.2 Serum biochemical indexs

Fasting glucose, fasting insulin, insulin resistance index, uric acid, and blood lipid level which includes total cholesterol, triglyceride, low-density lipoprotein, high-density lipoprotein will be measured on week 0, 8.

##### 2.5.2.3 Blood pressure

Blood pressure will be measured on week 0, 8.

##### 2.5.2.4 Conversion score of constitution characteristics

The constitution characteristics in the Table of Constitution Classification and Judgment of Traditional Chinese Medicine will be adopted as the criterion of constitution determination. The conversion score of constitution characteristics will be then calculated based on the computational formula: Conversion score= [(original score-number of entries)/(number of entries × 4)] × 100. Measurements will be made on week 0, 8, 12.

##### 2.5.2.5 Measurement of the scale

The IWQOL—Lite scale, and Emotional and Psychological Assessment scale will be measured on week 0, 8, 12. The lower the total score, the lighter the clinical symptoms of the patient.

### 2.6 Study procedure

Before study commencement, baseline demographic data and clinical characteristics will be recorded. Next, all the participants will complete a general information form, and then written informed consent will be provided to them. Participants who meet the inclusion criteria and exclusion criteria will be enrolled, grouped, and given corresponding interventions. The schedule of enrolments, allocation, and assessments is given in Table 1.

**Table 1:**
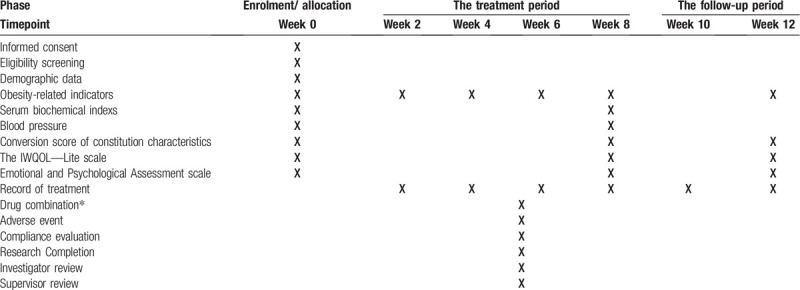
The schedule of enrolments, allocation, and assessments.

### 2.7 Sample size calculation

The participants will be randomly divided into moxibustion combined with characteristic lifestyle intervention of TCM (intervention group), moxibustion combined with lifestyle intervention (intervention group), the lifestyle intervention (control group) in a 2:2:1 allocation ratio. The sample size was calculated based on the primary outcome, which is proposed to be the WC. Statistical Analysis System software (version 9.3)(SAS 9.3 Institute Inc., Cary, NC) was used to calculate the required sample size.

Based on the results of previous studies and related literature reports, sample size required for each individual hypothesis testing was estimated at the 1-sided α level 0.025. With other assumptions, the basis of estimated sample sizes was listed in Table 2. To ensure adequate power for each individual hypothesis testing, a sample size of 135 was required, which consists of 54 subjects in the moxibustion combined with characteristic lifestyle intervention of TCM (intervention group), 54 subjects in the moxibustion combined with lifestyle intervention (intervention group), and 27 subjects in the lifestyle intervention (control group). Anticipating a 10% dropout rate, a total of 150 participants were recruited.

**Table 2:**
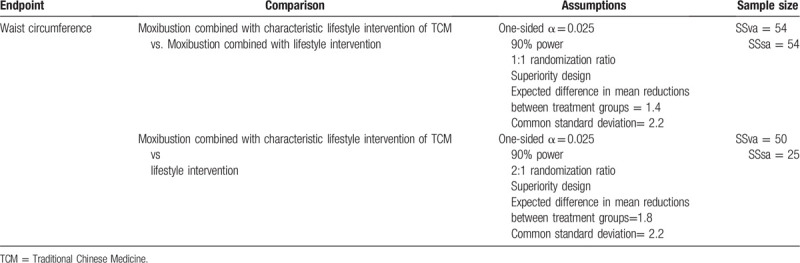
Sample sizes required for each individual hypothesis testing.

### 2.8 Randomization and blinding procedures

Eligible participants will be allocated to the 3 groups in a 2:2:1 ratio by a computer according to the central simple completely random grouping method of the SAS package (SAS statistical software version 9.3; SAS 9.3 Institute Inc., Cary, NC). The random scheme was compiled and produced by random statisticians in the data center of Chinese Academy of Chinese Medical Sciences, and generated through SAS software PROC PLAN process statement. The random number assignment will be generated by an independent statistician who will not be a member of the research team. Eligible subjects who have written an informed consent will be randomly assigned to one of these 3 groups. All the researchers who evaluate the results above will not be aware of which group the participants have been assigned to. All processes will be recorded and saved as appropriate.

### 2.9 Data collection and management

Clinical observation results such as the demographic data of the participants, clinical information, and any other data related to this study will be documented on case report forms (CRFs). Participants will receive a food and exercise diary and will be asked to complete from baseline to the end of the follow-up period. The diary will be collected every 2 weeks separately from each hospital. A corresponding computer database will be established and the medical records of this clinical trial will be input and stored by researches in a password-protected electronic database, and be kept strictly confidential. A member in the study team who is not involved in these data collection will check the database data and source documents. The hard copies of all the clinical data and CRFs will be stored in a fixed locker.

### 2.10 Statistical analysis

The SPSS software v19.0 will be used for statistical processing. The data of all participants will be conducted in the final statistical analysis which will be in accordance with the per-protocol principle and the intent-to-treat principle. Measurement data will be presented in the form of mean ± standard deviation, whereas count data will be expressed as percentage and number.

The homogeneity test of variance and normal distribution test will be adopted for measurement data. Count data and grade data will be described by rate and composition ratio. Student *t* test will be used when the data obey the normal distribution, whereas the data not obeying the normal distribution will use rank sum test. Comparison is performed by *χ*^2^ test, repeated measurement data are expressed by mean ± standard deviation and intra-group comparison is performed by analysis of variance of repeated measurement data, and inter-group comparison is by multivariate analysis of variance (MANOVA). All statistical tests will be performed on both sides, and the difference will be considered statistically significant when *P* < 0.05.

## 3 Discussion

The prevalence of overweight and obesity in 18 years or older population is about 72.5% in Mexico, whereas the prevalence of abdominal obesity almost reaches 76.6%, according to the Half Way National Survey of Health and Nutrition (ENSANUT MC) 2016, and this frequency is expected to increase.^[26]^ Abdominal obesity has been closely linked to insulin resistance, inflammation, full-blown diabetes, or blood lipid disorders, and even an increased risk of developing metabolic syndrome, cardiovascular disease, Alzheimer disease.^[27–29]^ Abdominal obesity has been considered to be the most influential cause of chronic liver disease which is associated with non-alcoholic fatty liver disease.^[30]^ Both abdominal obesity and general obesity have been regarded as the major public health concerns in the world. Also, a large economic burden caused by obesity has been imposed on the individual, families, and even nations.^[31]^ The global economic impact of obesity was estimated to be US $2.0 trillion or 2.8% of the global gross domestic product in 2014.^[32]^ Therefore, it is important to discover cost-effective therapy for obesity, especially abdominal obesity.

During the past 40 years, it has been illustrated that abdominal obesity is mainly caused by a dysfunction between the kidney, spleen, and the triple energizer in Chinese medicine research, and its pathogenesis is closely associated with many dysfunctional mechanisms of these visceral organs, with the involvement of stagnation of phlegm. We designed this study to examine the safety and efficacy of moxibustion combined with characteristic lifestyle intervention of TCM in the treatment of abdominal obesity. The patient's obesity-related indicators, serum biochemical indexs, blood pressure, conversion score of physical symptoms, and measurement of the scale will be recorded and reported.

As one of the most important traditional medical techniques in China, moxibustion has certain advantages in treating metabolic disorders. In this study, characteristic lifestyle intervention of TCM is comprised of xiusheng decoction, traditional exercises, and modern lifestyle intervention which have been commonly used in the treatment of abdominal obesity; all of them have gradually become the research hotspots in recent years. Li et al conducted a randomized controlled trial in 2018, which recruited 60 obesity patients with phlegm dampness stagnation syndrome and showed that warming needle moxibustion had positive effects on controlling clinical symptoms in these patients.^[33]^ Another randomized control trial in 2012 used a combination treatment of acupuncture and moxibustion in obesity patients at a frequency of 3 times per week for 12 weeks and found that there was significant difference in results between the control group and the treatment group.^[34]^ The study concluded that significant effects were observed since the symptoms improved after the intervention of acupuncture and moxibustion in the treatment group.^[34]^ Collectively, these effects could be the underlying impact mechanism of moxibustion on obesity. This hypothesis will be further validated in our study by the fact that moxibustion with characteristic lifestyle intervention of TCM was more effective than either moxibustion plus lifestyle intervention or lifestyle intervention alone in addressing the symptoms of abdominal obesity.

Among many of the mechanisms of abdominal obesity, chronic inflammation, cell apoptosis, energy imbalance, and gut microbial enterotypes can result in an excessive accumulation of adipose tissue.^[35,36]^ It is possible that moxibustion treatments could lead to the inhibition of inflammatory, promotion of energy metabolism, and reduction of pro-inflammatory cytokines. It has been shown that the clinical effects of moxibustion may encompass neural modulation, anti-inflammatory, immunity enhancement, and acceleration effects of the metabolism.^[37]^ A number of studies showed that moxibustion had some positive effects on the obesity-related parameter in abdominal obesity patients; however, insufficient evidence on moxibustion temperature, and treatment duration made results difficult to interpret. In addition, lifestyle changes (exercise, diet control) can reduce anthropometric indexes in patients with abdominal obesity and these interventions are focused on improving fat consumption and metabolism capacity.^[38]^ Also, moxibustion and lifestyle changes have many other advantages, including low-cost, stable efficacy, and low risks of complications. Although some studies confirmed such beneficial effects of moxibustion or lifestyle changes in improving abdominal obesity symptoms, the combined effects of moxibustion, and characteristic lifestyle intervention of TCM which includes lifestyle changes on abdominal obesity still remains unclear.

In this study protocol, we will use moxibustion at 4 acupoints for the treatment period of 8 weeks combined with characteristic lifestyle intervention of TCM for 12 weeks which also includes the follow-up period of 4 weeks. The outcome evaluations will include analyses of obesity-related indicators, serum biochemical indexs, blood pressure, conversion score of physical symptoms, and measurement of the scale. Besides, some limitations still exist in this proposed study. First, the effects of one 8-week treatment session will be observed and the effects of 4 time points will be mainly focused on during the intervention period. Because our purpose in this study is mainly to evaluate the safety of the major treatment and longer-term effects on improving obesity-related clinical symptoms, a relatively long treatment period will be required to determine the longer-term effect of this combined therapy and whether it is acceptable and safe for patients to use frequently. Secondly, the sample size is not enough and the blinding method cannot be completely used for the moxibustion therapist which will result in a bias risk of this clinical trial to some extent. Nevertheless, we expect that this study will be able to provide some new evidence regarding the combined effect of moxibustion and characteristic lifestyle intervention of TCM in the treatment of abdominal obesity.

## Acknowledgments

The authors extend their appreciation to all the patients who participated in this study. The authors are grateful for the support for this study: trial coordinating team, research departments, and statistical analysis staff.

## Author contributions

Li-Hua Wang, Wei Huang, and Wei Zhou designed the study protocol and contributed equally to the manuscript. Li Zhou, Xiao-Li Zhou, Peng Zhou, and Yan Yan reviewed the study protocol and drafted the manuscript. Li-Hua Wang is responsible for the sample size calculation and statistical analysis. Zhong-Yu Zhou, and Hua Wang contributed to the discussion. All authors carefully read and approved the final manuscript.

**Conceptualization:** Xiao-Li Zhou.

**Investigation:** Li Zhou, Peng Zhou, Yan Yan.

**Methodology:** Wei Huang, Wei Zhou.

**Writing – original draft:** Li-Hua Wang, Zhong-Yu Zhou.

**Writing – review & editing:** Hua Wang.
